# First Nations, Inuit and Métis Peoples Living in Urban Areas of Canada and Their Access to Healthcare: A Systematic Review

**DOI:** 10.3390/ijerph20115956

**Published:** 2023-05-25

**Authors:** Simon Graham, Nicole M. Muir, Jocelyn W. Formsma, Janet Smylie

**Affiliations:** 1 Department of Infectious Diseases, Peter Doherty Institute for Infection and Immunity, University of Melbourne, Melbourne, VIC 3010, Australia; 2 Psychology Department, York University, Toronto, ON M3J 1P3, Canada; 3 National Association of Friendship Centres, Ottawa, ON K2P 0L9, Canada; jwformsma@nafc.ca; 4 Dalla Lana School of Public Health, University of Toronto, Toronto, ON M5T 3M7, Canada; 5 Well Living House, and Department of Family and Community Medicine, St. Michael’s Hospital, Toronto, ON M5B 1W8, Canada

**Keywords:** Indigenous, barriers, facilitators, discrimination, culture, healthcare

## Abstract

In Canada, approximately 52% of First Nations, Inuit and Métis (Indigenous) peoples live in urban areas. Although urban areas have some of the best health services in the world, little is known about the barriers or facilitators Indigenous peoples face when accessing these services. This review aims to fill these gaps in knowledge. Embase, Medline and Web of Science were searched from 1 January 1981 to 30 April 2020. A total of 41 studies identified barriers or facilitators of health service access for Indigenous peoples in urban areas. Barriers included difficult communication with health professionals, medication issues, dismissal by healthcare staff, wait times, mistrust and avoidance of healthcare, racial discrimination, poverty and transportation issues. Facilitators included access to culture, traditional healing, Indigenous-led health services and cultural safety. Policies and programs that remove barriers and implement the facilitators could improve health service access for Indigenous peoples living in urban and related homelands in Canada.

## 1. Introduction

### 1.1. First Nations, Inuit and Métis Peoples

First Nations, Inuit and Métis (Indigenous) peoples are the recognized Indigenous peoples in Canada [1]. Each has their own colonial history, and there is diversity and relatedness within and between these distinct peoples [1].

### 1.2. The Migration of Indigenous People to Urban Areas

Indigenous peoples in Canada are becoming more urbanized, with the 2016 census highlighting that 52% of First Nations, 62.6% of Métis and 56.2% of Inuit peoples lived in urban areas [2]. From 2006 to 2016, the estimated number of Indigenous peoples living in urban areas increased by 59.7% [2].

Métis people have been living in and migrating to Canadian cities since the founding days of the Métis nation [3]. In 1951, amendments to the Indian Act repealed a law that limited the free movement of First Nations peoples off reserves, resulting in the migration of First Nations people to urban areas [4]. The Indian Act also contributed to First Nations women’s migration to cities because if a First Nations woman with status (formally recognised as a First Nations woman) married a non-status man, she would lose her status and therefore be unable to live on reserve; which forced many First Nations women to move to urban areas [5]. The federal government began to actively implement polices of Inuit relocation from traditional territories to permanent settlements in the Nunangat or southern urban centres during WWII [3,4,5]. Food insecurity and access to health care, housing, employment, and education have prompted the ongoing migration of Inuit people to urban centres [3,6].

### 1.3. Access to Health Services in Urban Areas of Canada

Compared to remote areas of Canada, urban centres have a higher per capita density of primary healthcare, mental health, social support and specialist health services. Despite this urban concentration of services, Indigenous peoples living in urban areas do not necessarily have better health care experiences and treatment outcomes compared to First Nations people living on reserves or Inuit people living on Nunangat. The First Nations Regional Health Survey (RHS) found that 21.3% of First Nations people living on-reserve reported not having a primary health care provider, and 9.6% reported unmet health needs in the previous 12 months [7]. In the urban city of Toronto, 37.4% of Indigenous people reported not having a primary health care provider, and 28.0% reported having unmet healthcare needs in the previous 12 months [8]. This highlights that some Indigenous peoples living in urban areas, despite living closer to a range of health services, are having difficulty accessing health care services or are choosing not to access these services.

### 1.4. Aim

This study aims to highlight the barriers Indigenous people experience and the facilitators that could improve access to health services among Indigenous peoples living in urban areas of Canada.

## 2. Materials and Methods

### 2.1. Conducting the Review

This systematic review was conducted according to the Preferred Reporting Items for Systematic Reviews and Meta-Analyses: The PRISMA Statement [9] but is not registered with the International Prospective Register of Systematic Reviews (PROSPERO). The primary research question was the following: What are the barriers to and facilitators of access to health services for First Nations, Inuit and Métis peoples living in urban areas of Canada?

### 2.2. Search Strategy

We searched the electronic databases Embase, Medline and Web of Science from 1 January 1981 to 30 April 2020 using the following MeSH terms and variations:

1. (First Nations OR Inuit OR Métis OR Indigenous OR Aboriginal OR Native) AND
2. (Canada) AND
3. (Urban OR urbanized OR city OR cities OR metropolitan) AND
4. (clinic OR medical OR doctor OR nurse OR physician OR primary health service OR mental health OR hospital OR drug use services) AND
5. (access OR accessing)

### 2.3. Inclusion Criteria

Reference lists of included studies were examined for additional studies. We also searched for relevant grey literature including government or community reports using Google. Studies were included if they had a focus on First Nations (status or non-status), Inuit or Métis peoples who lived in urban areas of Canada and provided information about barriers or facilitators to accessing health services. Studies that reported combined results of Indigenous people living in urban and rural/remote settings were included if there was content specific to urban health care.

### 2.4. Exclusion Criteria

Studies were excluded if they were guidelines, reviews, opinion pieces, not in English, contained no Indigenous specific results, or the full paper could not be accessed electronically or through author communication. If a study met the inclusion criteria but it did not specify the distinct Indigenous nations included in the study (i.e., First Nations, Inuit or Métis) then the authors labelled the study population as ‘Indigenous’, recognizing that pan-Indigenous approaches that combine distinct Indigenous populations have limitations. In alignment with Statistics Canada, urban areas were defined as a population of ≥1000 and a population density of >400 persons/km [10].

### 2.5. Data Extraction

The first author reviewed each study using Covidence software [11] and removed studies that did not meet inclusion criteria. The first and second authors then worked together to reach consensus on the included studies. Data extraction was conducted by the first author and inserted into an excel spreadsheet [12]. The following information was extracted: author, year the study was published, years the study was conducted, city and province, Indigenous group (First Nations, Inuit, Métis), study design, how the sample was chosen, sample size, population group (adults, youth, Elders), type of health service (general practitioner, emergency department of a hospital, substance use service, dental clinic, etc.), incentives provided (cash, gift cards), aim of the study, barriers to health service access and facilitators to health service access.

### 2.6. Ethics

This study did not seek ethics approval because it used publicly available studies and government and community reports. It was reviewed and approved by the board of an Indigenous organization of which the third author is a senior staff member.

## 3. Results

### 3.1. Overview of Included Studies

**Figure 1:**
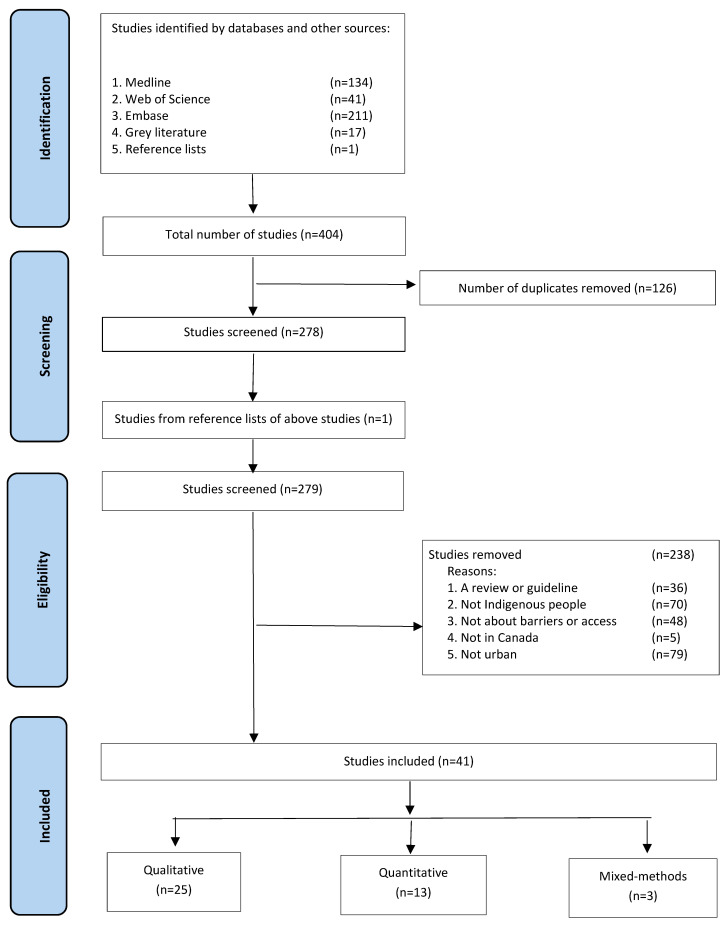
Flow diagram of included studies.

Overall, 41 studies met the inclusion criteria (Figure 1 and Table 1) [13,14,15,16,17,18,19,20,21,22,23,24,25,26,27,28,29,30,31,32,33,34,35,36,37,38,39,40,41,42,43,44,45,46,47,48,49,50,51,52,53].

### 3.2. Barriers of Accessing Health Services

Barriers of accessing health care services for Indigenous peoples living in urban areas included difficult communication with health professionals, medication issues, dismissal by healthcare staff, wait times, mistrust and avoidance of healthcare, racial discrimination, poverty and transportation issues (Table 2 and Supplementary Table S1).

#### 3.2.1. Difficult Communication with Health Care Professionals

Difficult communication with hospital staff was highlighted as a barrier [28,37,41,43,52,53]. Participants gave examples of when they were in medical facilities and not listened to, not believed or spoken to in a condescending manner [28,35,42]. Some participants spoke of not understanding what the healthcare providers were saying to them. When a family member accompanied one participant to the hospital, the participant said the following:

*This time it was not as bad because my daughter came with me. I felt I was treated alright… I felt this time around the staff treated me good and this time I understand as the doctor talked slow to me and when I don’t understand the question I asked him to explain it to me better. I feel more comfortable now*.

[53]

One participant who was pregnant felt she did not have time to ask questions and said the following:

*The doctor himself is so abrasive—flies into the room, does what he needs to do … it doesn’t really seem like he cares, and he is out the door and on to the next patient. … I feel so rushed that I don’t actually get to talk about things that are pertinent to my pregnancy. And so I leave the office and did not voice my concerns.*

[27] (p. 5)

Finally, one study highlighted a fear that disclosing spiritual gifts to non-Indigenous healthcare providers could bring about a mental health misdiagnosis [25], suggesting that Indigenous patients may not feel safe disclosing traditional cultural practices to healthcare providers.

#### 3.2.2. Medication Issues

Some participants who had a history of substance use were denied medications for telling the truth about their substance use [20,34,35]. Two studies which included people who used illicit drugs highlighted that when they told the truth about their drug use, they were kicked out of the doctor’s office or were told that they needed a clean urine test before they could receive their medications [20,35]. A non-Indigenous nurse acknowledged that “*there is a systemwide belief that Indigenous peoples misuse pain medications, and as a result, Indigenous peoples are not provided adequate pain medication*” [48] (p. 43).

One study participant stated that her doctor did not know that she should go on medication for her condition, and she had to educate him [33]. There was an example of fear towards being overprescribed medications with one participant reporting that they were terrified of medication due to seeing a family member being heavily medicated because of mental health issues [25].

#### 3.2.3. Dismissal or Discharge by Healthcare Staff

Indigenous people living in urban areas described being either verbally or physically dismissed from healthcare facilities and how this affected their ability to access healthcare including instances where they avoided/delayed seeking healthcare until they were very sick. Examples of dismissal include being threatened by hospital security or being involuntarily discharged from the hospital [16,20]. One participant said the following:

*My pneumonia hadn’t even [gone away] and it was during winter time. And …*

*one of the nurses came in and said the doctor is discharging you. I said I’m not even better yet and she said, well it’s time for you to go … don’t let me call security. And sure enough she called security. Security literally came in, grabbed me behind my arms, dragged me down the hallways and threw me out the door, with pneumonia, in winter time.*

[16] (p. 1112)

#### 3.2.4. Wait Times

Studies highlighted that Indigenous people spend a lot of time waiting for healthcare services. Participants in Barnabe and colleagues’ (2017) study said that they had difficulty having the physician make a referral, obtaining an appointment once referred and waiting too long to see the referral physician as the referral appointments were often cancelled or deferred. Other participants reported that there were long wait lists to access healthcare in general, long waiting times once they were in the doctor’s office or emergency room and long waits for test results [19,27,33,34,42,49,53]. Another participant, highlighted how long waiting times discouraged them from seeking timely assessment:

*I notice every time I go see a doctor, I’m waiting for a long time. Like my knee, I handled that for about a week and a half before I even decided to go [for treatment] because I knew the waiting time was just going to be a long time*.

[42] (p. 705)

#### 3.2.5. Mistrust and Avoidance of Healthcare

Studies highlighted the mistrust that urban Indigenous people felt towards the health care system [53]. Previous experiences of accessing health services contribute to Indigenous mistrust of healthcare providers [19,34]. One Indigenous Elder from Schill and colleagues’ (2019) study commented that

*Sometimes we don’t trust the doctors … because we don’t know what they’re going to give us. And sometimes that can harm our body … That’s why when I was smoking and I was coughing for three days, I didn’t go to the hospital because I’m scared of hospitals. Sometimes it’s trust.*

[39] (p. 871)

#### 3.2.6. Racial Discrimination

Studies identified racial discrimination as a barrier to accessing health services [15,29,30,39,41,43,48]. One study interviewed health care providers about Indigenous patients with one healthcare professional stating that “*there are times when Indigenous patients come in with expectations of poor treatment, which sets the stage for a challenging interaction*” [49]. A doctor also said, “*as a physician, sometimes I become defensive when an interaction with an Indigenous patient is not going well*” [48] (p. 40). Health professionals acknowledged their lack of understanding of Indigenous issues and culture, and that their views about Indigenous people were informed by the media.

Participants highlighted that being Indigenous elicited anti-Indigenous discrimination [20,37,41,52]. One participant noted that

*The healthcare workers treated me like crap and I know it was because I was Native … When you need the medical care we put up with it. We shouldn’t have to*.

[20]

Studies highlighted the harms that discrimination can have on an individual’s health-seeking behaviours [18,40]. The Our Health Counts Toronto study found that 71% of Indigenous adults living in Toronto reported experiencing racism from healthcare professionals which then prevented, stopped or delayed them from returning to seek healthcare [45].

#### 3.2.7. Poverty and Transportation

Poverty was identified as a barrier even within Canada’s universal health care system [16,19,20,37,43,49,52].

Indigenous peoples were concerned with how healthcare providers might be responding to them based on their appearance as people living in poverty and poor neighbourhoods [16,52].

One participant noted that

*… you have to expect living in this area you’re not going to get the best healthcare. It seems like they care less when you‘re in a poverty-stricken area … the doctor’s office is kind of ghetto looking … It doesn’t feel personable, it doesn’t feel welcoming, and it feels like you’re in and out, and they are not doing their job. They don’t ask you how you’re doing, as they would in a different nicer area*.

[47]

Poverty also includes not being able to afford to take transportation to medical appointments and was mentioned in four studies [19,27,46,52]. One study interviewed an Indigenous pregnant woman who noted that

*“I was supposed to go for an ultrasound, but I couldn’t go. It was cold that day and I wasn’t gonna walk. I didn’t have a bus fare … didn’t want to freeze my ears, so I just stayed home”*.

[27]

### 3.3. Facilitators of Accessing Health Services

The facilitators to accessing health care were access to culture, traditional healing, Indigenous-led healthcare services and support around their needs such as food and transportation (Table 2 and Supplementary Table S1).

#### 3.3.1. Access to Culture

The opportunity to use and practice culture was highly valued by Indigenous peoples living in urban areas [13,14,22,25,36]. One participant reported that, “*to have wellness, it means having access to your culture and to resources and support*” [25] (p. 94). Community-designed teachings, traditional healing services, services in different Indigenous languages (e.g., Oji-Cree, Cree, Inuktitut, Ojibway), medicine walks, dancing, drumming, traditional arts and crafts, sweat lodges and ceremonies were available and highly valued as part of their healthcare [13,14]. Access to and use of traditional languages was also identified in several studies as a facilitator of healthcare access [13,14,22,44].

There was recognition of mainstream medicine and its benefits, but this was commonly in a context in which participants identified that they had access to both mainstream and traditional Indigenous health and wellbeing practices (e.g., naturopaths, social workers) [36]. One participant remarked that

*“I do see a clinical counsellor every couple of weeks but I don’t see that as being more helpful than going to the beading group, than going to Métis Night at the Friendship Centre”.*

[25] (p. 95)

#### 3.3.2. Traditional Healing

Access to traditional healing was identified as highly important in multiple studies. The personal connection with Traditional Healers was cherished by Indigenous people in these studies [13,17,23,36]. One Indigenous person noted that

*Doctors today don’t know who we are, especially when we are using walk-in clinics. Our traditional doctors knew us, they knew our family, and they talked to our ancestors in ceremony. If we got sick, our parents knew where to go, and not just to one person, there were different people in the community.*

[36] (p. e395)

#### 3.3.3. Indigenous-Led and Run Health Services

Grey literature provided valuable information regarding Indigenous community-led and -run health services. These services improved access and connection to traditional healing alongside mainstream medical services [14,24,43]. A report by the Health Council of Canada (2012) described a range of projects designed and based in Indigenous communities. One example was Clinique Minowe which began in 2011 and included a nurse and a social worker providing home visits. Within the two years, the program significantly increased access of a broad range of health, social services and programs. Staff built trust through a visible and active presence in the community. Families who have an established relationship with Clinique Minowe were more likely to attend their western medical or social service appointments compared to before the clinic was set up [24].

Studies described the higher quality of care at Indigenous-led and -run health services [39,44,46,51]. One participant commented that

*I went to another downtown clinic and the doctor that I had was giving me constantly the same pills all the time when I was getting sick. I went over to the Native Health and the doctor there, as soon as she saw me, said, ‘Get to the hospital.’ And now she is my doctor. She is somebody who cares and takes the time to listen to me.*

[51] (p. 826)

#### 3.3.4. Access to Culturally Safe Care

Cultural safety was identified as a high priority that increased access, including return visits to health services, especially in one study where a participant said the following:

*I think that I have to mention cultural safety. It’s so important. It’s something that should be a way of being for everyone, so that we can develop respectful relationships with no matter who it is. […] If I know where our people are, like the Ki-Low-Na Friendship Society, I’d rather go there.*

[39] (p. e827)

## 4. Discussion

This study highlights the main barriers to health services for Indigenous people living in urban areas and identified facilitators that could improve Indigenous people accessing health services. Although this study focused on the perspectives and experiences of Indigenous peoples, there was one study that included the perspectives of health professionals when providing services to Indigenous peoples in urban areas.

Only one study focused on Métis peoples [25] and Inuit peoples [43], respectively. Additional studies that included Métis and Inuit peoples also included First Nations peoples. Just under half of the studies in our analysis included First Nations peoples only. The three distinct Indigenous groups seemed to face similar challenges of racial discrimination and negative interactions with health professionals. This highlights the need for increased education among health professionals about the three distinct Indigenous groups in Canada.

Studies described tensions between Indigenous peoples and health professionals. The organizational practices within institutions such as hospitals and primary health services can impact health professionals’ behaviour towards Indigenous people. In recent years Indigenous leaders and communities have advocated for health institutions to implement cultural safety training with their staff. Cultural safety is a concept developed by Māori nurse Irihapeti Ramsden in the 1990s in response to the way Māori people were being treated by health institutions [54,55]. Her work proposes three steps towards culturally safe practices: firstly, cultural awareness or understanding of differences; secondly, cultural sensitivity where people accept the legitimacy of difference; and thirdly, reflecting on the impact of the service provider’s life experience and positioning on others. In Canada, a team in Montreal implemented cultural safety training with 45 nurses, social workers and doctors. The program was successful at raising awareness of Indigenous culture and challenges Indigenous peoples face when accessing health services, the importance of including Elders in the design and delivery of services and to decolonize health care systems [56]. Similar to New Zealand and Canada, Indigenous leaders in Australia have advocated for cultural safety training. A report by McDermott and colleagues highlights why institutions should imbed cultural safety as an ongoing program for all health care staff to reduce racism, raise awareness and increase access to health services for Indigenous peoples [57]. A review in Australia found that the large disparity in health outcomes between Indigenous and non-Indigenous people could be attributed to institutional racism and intergenerational trauma [58,59]. From an institutional perspective, cultural safety training may provide a step forward to improving Indigenous peoples experiences with health professionals.

In our review, the study by Wylie and colleagues (2019) suggests that stereotypes within the health profession regarding Indigenous peoples set the stage for challenging interactions. This aligns with literature that highlights “cultural differences”; nurses ‘othering’ Indigenous peoples; and assumptions about Indigenous peoples that influenced clinical practice [60,61]. The cultural safety approach taken by Indigenous leaders in Canada, Australia and New Zealand could provide some useful ways health care institutions could reduce racism and increase access to health services for racial minorities in these developed nations. In the United Kingdom, the National Health Service is attempting to reduce challenges black and Asian populations face when accessing the National Health Service (the United kingdom’s universal health system). Using the approaches Indigenous leaders have taken may provide a starting point to developing similar cultural safety training to achieve these reductions in discrimination and raise awareness [62].

Access to Indigenous-led and -run health services was highly valued by Indigenous peoples. These community-based health services are commonly rooted in Indigenous ways of knowing and doing; Indigenous community, identity and inclusion; and Indigenous culture and cultural protocols—all of which can contribute to cultural safety. Two studies highlighted the importance of these more inclusive ways of approaching health and wellbeing and linked implementation strategies including equity-focused organizational structures, policies and processes; contextually tailored care; and culturally safe spaces for Indigenous patients [44,52].

Holistic healthcare that includes a person’s emotional, mental and physical wellbeing was identified as important to Indigenous peoples [13,14,43]. The concept of holistic healthcare and wellbeing has been widely acknowledged as having benefits [58]. There is some evidence supporting the benefits of holistic wellbeing programs, particularly for Indigenous peoples who have spent time in prison and then re-enter the community [63]. Continued efforts to look beyond the one health issue for which an individual is attending a health service are needed. This indirectly results in other health conditions being identified early even though the patient may not have attended the health care service for that health issue. Programs in Australia that provide financial incentives to Indigenous health service providers upon completion of general preventative “health checks” have shown good uptake [64]. These adult health checks aim to advance the wellbeing of Indigenous peoples in Australia by conducting a range of general health checks to identify any health issue early and commence a plan to address it. This could be one way to provide holistic healthcare in Canada.

Access to traditional healing was also of high importance among Indigenous participants [13,14,32,37,43]. A study by Hossain et al. (2020) found that having a strong connection to your Indigenous culture seemed to have health benefits. A review by Asamoah and colleagues highlighted that in Canada, Australia and New Zealand, traditional healing was used in three ways: firstly, as the main choice of treatment; secondly, as an add-on option to western medical treatment; and thirdly, through adopting traditional knowledge within mainstream health care institutions [65]. This suggests that Indigenous people who move to urban areas for a range of reasons might benefit from connecting to Indigenous culture and with other Indigenous people living in urban areas.

### 4.1. Limitations

Limitations include quantitative studies that, while providing useful information, were not constructed to provide in-depth information regarding barriers and facilitators [19,29,30,46,49,52].

### 4.2. Future Research

There is a need for longer-term funding of Indigenous-led healthcare services and Indigenous child and youth services [41,45]. Funding is needed both for healthcare institutions to collaborate with Indigenous organizations and peoples to design and implement cultural training and for Indigenous organizations and peoples to evaluate these cultural safety programs. It is also important to provide continuing cultural awareness and education opportunities for non-Indigenous healthcare workers [29].

Future research needs to also advance Indigenous-led health information infrastructure for Indigenous peoples living in urban areas. For example, the various Our Health Counts studies have demonstrated limitations in estimating the true number of Indigenous peoples living in urban areas [66]. One component of this work includes linking cohorts of Indigenous peoples living in urban areas to provincial healthcare utilization datasets to address gaps in population-based information regarding healthcare usage. Regular reporting of primary and tertiary healthcare use for Indigenous peoples is essential to identify service gaps and to identify ways to reduce emergency room visits and hospitalizations. This work should be Indigenous-led and focus on the research questions that the community wants answered. Smylie and colleagues (2011) noted that “*self-determination is fundamental and thus [Indigenous] peoples must have full involvement and choice in all aspects of health care delivery, including governance, research, planning and development, implementation and evaluation*” [41] (p. 82).

Future research that examines the barriers Two-Spirit peoples face when accessing healthcare and the barriers and facilitators to healthcare for Indigenous Elders living in urban areas is also needed [22].

## 5. Conclusions

Indigenous people living in urban areas are experiencing barriers in healthcare access. A history of discrimination is negatively influencing interactions between non-Indigenous health professionals and Indigenous peoples. Practical ways to implement the facilitators is a way forward to increasing access. Indigenous-led research that meets community needs should be encouraged. Additionally, providing and evaluating cultural safety and awareness training to non-Indigenous healthcare providers is needed.

## Figures and Tables

**Table 1:** Studies examining health service access among First Nations, Inuit and Métis peoples living in urban areas of Canada.

Author, Year Published	Location	Indigenous Group ^A^	Participants	Sample Size	Study Design, Methods	Health Service Focus ^B^	Incentives Provided
Aboriginal Health Access Centres, 2015 [14]	Ontario	First Nations, Inuit, Métis	Adults	50,000	Quantitative	Primary health services	Not Stated
Aboriginal Health Access Centres, 2016 [13]	Ontario	First Nations, Inuit, Métis	Adults	50,000	Quantitative	Primary health services	Not stated
Auger et al., 2016 [36]	Vancouver, British Columbia	First Nations (status), First Nations (non-status), Métis	Family members or carers of Indigenous people with type 2 diabetes	35	Qualitative	Diabetic patients	$35 gift card
Auger, 2019 [25]	Vancouver, British Columbia	Métis	23 women and 10 men accessing mental health services	33	Qualitative	Mental health	$25 gift card
Barnabe et al., 2017 [46]	Calgary, Alberta	First Nations	Adults	38	Quantitative	Primary health services at the Elbow River Health Lodge	None provided
Beckett et al., 2018 [49]	Hamilton, Ontario	First Nations	Adults	524	Quantitative	Diabetic patients	$20 plus $10 for each person they recruit
Benoit et al., 2003 [51]	Vancouver, British Columbia	First Nations	Adult women	61	Qualitative	Primary health services at the Vancouver Native Health Society (VNHS) and *Sheway*	An honorarium was provided
Benoit et al., 2019 [50]	Toronto and Thunder Bay, Ontario	First Nations, Inuit, Métis	Women living with and without HIV	90	Mixed methods	Multiple	Not stated
Browne et al., 2011 [52]	Vancouver, British Columbia	First Nations, Métis, non-status Indigenous people	Patients of the emergency department (ED) and ED staff. 44 patients, 38 staff.	82	Quantitative	Emergency department of a hospital	None provided
Cameron et al., 2014 [53]	Edmonton, Alberta	First Nations, Métis	Aboriginal patients in hospital and their families	19	Qualitative	Emergency department of a hospital	Not stated
Carter et al., 2014 [15]	Vancouver, Victoria, Prince George, British Columbia	First Nations	Women living with HIV	28	Qualitative	HIV testing and treatment services and hospitals	Not stated
Denison et al., 2014 [16]	Northern British Columbia	First Nations, Inuit, Métis	Mothers where apprehension of their children is being threatened	9	Qualitative	Hospital	Not stated
Environics Institute, 2010 [17]	Vancouver, Edmonton, Calgary, Regina, Saskatoon, Winnipeg, Thunder Bay, Montreal, Toronto, Halifax and Ottawa	First Nations, Inuit, Métis	Adults	2614	Qualitative	Multiple	Not stated
Firestone et al., 2014 [19]	Hamilton, Ontario	First Nations	Adults	554	Quantitative	Primary health care services	$20 to participate plus $10 for each person they recruited
Firestone et al., 2015 [18]	Hamilton, Ontario	First Nations	Adults	554	Quantitative	Mental health	$20 to participate plus $10 for each person they recruited
Goodman et al., 2017 [20]	Vancouver, British Columbia	First Nations	Adults	30	Qualitative	Drug, alcohol and substance use services	Not stated
Goodman et al., 2019 [21]	Winnipeg, Manitoba	First Nations	Young people (15–25 years)	8	Qualitative	Primary health services	None provided
Health Council of Canada, 2003 [22]	Multiple provinces	First Nations, Inuit, Métis	Pregnant women and mothers	Not reported	Qualitative	Maternal, post natal and child health	Not reported
Health Council of Canada, 2012 [24]	Saskatoon, Winnipeg, Edmonton, Vancouver, Toronto, Montreal, and St. John’s	First Nations, Inuit, Métis	Adults	160	Qualitative	Primary health services	Not reported
Health Council of Canada, 2013 [23]	Vancouver, Winnipeg, Ottawa, Iqaluit, Inuvik, and Happy Valley-Goose Bay	First Nations, Inuit, Métis	Elders	Not reported	Qualitative	Primary health services	Not reported
Heaman et al., 2015 [27]	Winnipeg, Manitoba	First nations	Pregnant women	26	Qualitative	Maternal and prenatal care	$20 grocery gift card
Heaman, 2018 [26]	Winnipeg, Manitoba	First nations	24 postpartum women, 30 healthcare providers	24	Qualitative	Maternal and prenatal care	$20 grocery gift card
Hole et al., 2015 [28]	Kelowna, British Columbia	First Nations	Adults	28	Qualitative	Hospital	Not stated
Kitching et al., 2020 [29]	Toronto, Ontario	First Nations	Adults	836	Quantitative	Primary health service	$20 to participate plus $10 for each person they recruited
Lawrence et al., 2016 [30]	Ontario, Manitoba	First Nations, Métis	Pregnant women	541	Quantitative	Dental services	Not stated
Loyola-Sanchez et al., 2020 [31]	Southern Alberta	Indigenous	Patients needing Arthritis services	13	Qualitative	Rheumatology arthritis practices	Not stated
McCaskill et al., 2011 [32]	Toronto, Ontario	First Nations	Adults	1059 (623 surveys and 436 interviews)	Mixed methods	Multiple	$5 gift card
Mill et al., 2008 [33]	Vancouver, Edmonton, Winnipeg, Ottawa, Toronto, Montreal, Halifax, Labrador, Inuvik	Aboriginal	Youth (15–30 years)	441 (413 surveys and 28 interviews)	Mixed methods	HIV testing and management	Participants were provided with a small token of appreciation (no further info provided). Participating organizations received a small compensation for staff time.
Nelson et al., 2018 [34]	Prince George, British Columbia	First Nations	Adults	50	Qualitative	Primary health care service	Not stated
Nowgesic et al., 2015 [35]	Saskatoon and Prince Albert, Saskatchewan	First Nations	Adults	20	Qualitative	HIV treatment and management	Cash $20 per hour, travel expenses $20, childcare expenses $40, a small tobacco bundle, an Indigenous gift
O’Brien et al., 2016 [37]	London, Ontario	First Nations, Inuit, Métis	Adults	Not stated	Quantitative	Any type	$20 to participate plus $10 for each person they recruited
Pearce et al., 2019 [38]	Vancouver, Prince George, Sudbury, Regina, Saskatchewan	First Nations, Inuit, Métis	People who use illicit drugs and are accessing hepatitis C treatment	45	Qualitative	Hepatitis C clinics	Cash
Schill et al., 2019 [39]	Kelowna, British Columbia	First Nations, Inuit, Métis	Elders	9	Qualitative	Mental health	$25 for each sharing circle the elders attended
Smylie et al., 2011 [41]	Hamilton, Ontario	First Nations, Inuit, Métis	Adults and children	790	Quantitative	Any type	$10
Syme et al., 2011 [40]	Vancouver, British Columbia	First Nations, Inuit, Métis	Adults	60	Qualitative	Mental health and addictions services	$30 each
Tang et al., 2015 [42]	Vancouver, British Columbia	Indigenous	Adults	34	Qualitative	Emergency department of a hospital	Not stated
Tungasuvvingat Inuit, 2017 [43]	Ottawa, Ontario	Inuit	Adults	345	Quantitative	Any type	$10 to participate plus $10 for each person they recruited
Van Herk et al., 2012 [44]	Ottawa, Ontario	First Nations, Inuit, Métis	Adults	26	Qualitative	Services for women, social services for all	Not stated
Well Living House, 2016 [45]	Toronto, Ontario	First Nations, Inuit, Métis	Adults	Not stated	Quantitative	Any type	$20 to participate plus $10 for each person they recruited
Wright et al., 2019 [47]	Hamilton, Ontario	First Nations, Métis	Pregnant women	19	Qualitative	Primary health services	Not stated
Wylie et al., 2019 [48]	Urban city in southern Ontario	First Nations, Inuit, Métis	Health care providers	25	Qualitative	Primary health services	Not stated

^A^—Indigenous group: the Indigenous group reported by each study is used; if the study did not report a specific group, it was labelled as ‘Indigenous’. ^B^—Health service type: Emergency department of a hospital, mental health service, primary health care service, dental services, addiction and substance use services, maternal health.

**Table 2:** Barriers and facilitators of accessing health services among First Nations, Métis and Inuit peoples living in urban areas of Canada.

Author, Year	Barriers to Accessing Health Care	Facilitators to Accessing Health Care
Aboriginal Health Access Centres, 2015 [14]	Not the focus of this study	Holistic wellbeing Culture as treatment Indigenous community led and run primary health services
Aboriginal Health Access Centres, 2016 [13]	Not the focus of this study	Indigenous knowledge of the life cycle Indigenous concept of holistic health The continuity of care from health promotion and prevention to rehabilitation Integrating western medicine with traditional healers
Auger et al., 2016 [36]	Racism Discrimination Mistrust	High value of traditional healing that is based on relationships Increase in owning their own health and knowing about other options for health
Auger, 2019 [25]	Increased access to culturally responsive health care spanning both Western and traditional systems	Wellness was understood as a whole, including mental, emotional, spiritual and physical health
Barnabe et al., 2017 [46]	Long waiting periods Transportation issues Language	No discrimination Easy to access the service Good communication with patients
Beckett et al., 2018 [49]	Not having NIHB insurance Long waiting lists Income Poverty Not culturally appropriate	Not the focus of this study
Benoit et al., 2003 [51]	Difficult communication Poverty No traditional healers	Good communication Holistic wellbeing approach Indigenous health services
Benoit et al., 2019 [50]	Racism, negative stereotypes and judgements Sixties Scoop The Indian Act Indian Residential Schools Inadequate health services Socio-economic insecurity A lack of recognition of Indigenous knowledge systems	Not the focus of this study
Browne et al., 2011 [52]	Discrimination Difficult communication with staff	Caring about the person Holistic wellbeing approach
Cameron et al., 2014 [53]	Limited access to specialized care Long waiting times Barriers in the communication and understanding of medical jargon Barriers in the interaction with health care professionals	Good communication with health professionals Having a family member go with you
Carter et al., 2014 [15]	Racism, discrimination	Not the focus of this study
Denison et al., 2014 [16]	Racism, discrimination	Not the focus of this study
Environics Institute, 2010 [17]	Not the focus of this study	Traditional healing, easy access to services
Firestone et al., 2014 [19]	Transportation Doctor not being available Low income and poverty Services not covered by Non-Insured Health Benefits Lack of trust in healthcare provider	Not the focus of this study
Firestone et al., 2015 [18]	Discrimination	Not the focus of this study
Goodman et al., 2017 [20]	Participants’ experienced medical dismissal often which resulted in disengagement from care or delay in care	Good communication with health professionals Holistic wellbeing approaches
Goodman et al., 2019 [21]	Residential mobility Racism negatively influenced the types of social support and relationships formed Improved access to health-promoting social programs	Culturally based services
Health Council of Canada, 2003 [22]	Not the focus of this study	Traditional healing Indigenous community-based services
Health Council of Canada, 2012 [24]	Not the focus of this study	Welcoming Feeling culturally safe Feeling like you belong
Health Council of Canada, 2013 [23]	Isolation Poverty	Consulting with and building equal partnerships with First Nations, Inuit and Métis peoples Dedicated Indigenous health centres and case managers Whole of community programs to assist seniors and elders, e.g., Peter Ballantyne Cree Nation, Kahnawake Shakotiia’takenhas Community Services, Saanich First Nations Adults Care Society Increased use of Telehealth Acknowledging and integrating traditional culture into care services
Heaman et al., 2015 [27]	Transportation Childcare Could not afford transportation to get to prenatal appointments Long periods in a waiting room Negative personality of PNC staff, such as being rude or abrasive, distracted, or not caring	Transportation assistance Convenient location of services Positive care provider qualities Women were motivated to attend prenatal care to gain knowledge and skills and to have a healthy baby
Heaman, 2018 [26]	A better understanding of other programs arising from their involvement Improved communication Benefits of teamwork Positive changes in service delivery (e.g., more accessible, convenient)	Convenient Good communication
Hole et al., 2015 [28]	Structural violence that reproduces experiences of institutional trauma in hospital	Positive culturally safe experiences, were described as interpersonal interactions with feelings, being visible, being heard, being respected, treatment as a “human being”
Kitching et al., 2020 [29]	Racism and discrimination	Not the focus of this study
Lawrence et al., 2016 [30]	Racism, discrimination	Not the focus of this study
Loyola-Sanchez et al., 2020 [31]	Good relationships with health providers	Increasing patient–provider trust. Patients’ narratives identified that patient–provider trust could be fostered by an environment that is safe, collaborative, and professional
McCaskill et al., 2011 [32]	Discrimination, Racism	Indigenous led and run services
Mill et al., 2008 [33]	Discrimination	Indigenous culturally based services, holistic wellbeing
Nelson et al., 2018 [34]	Lack of quality of care Long wait times Racism and discrimination	Not the focus of this study
Nowgesic et al., 2015 [35]	Accessing antiretroviral therapy within the context of living with a substance use disorder was an overarching theme	Not the focus of this study
O’Brien et al., 2016 [37]	Inability to obtain or afford transportation Poverty Lack of trust in health care providers	Not the focus of this study
Pearce et al., 2019 [38]	First: treatment providers must understand and accept colonization as a determinant of health and wellness among hepatitis C affected Indigenous people, including ongoing cycles of child apprehension and discrimination within the healthcare system Second: consistently safe attitudes and actions create trust within hepatitis C treatment provider–patient relationships and open opportunities for engagement into care Third: treatment providers who identify, build and strengthen circles of care will have greater success engaging hepatitis C affected Indigenous people who have used drugs into care	Good communication Respectful relationships Trust Holistic wellbeing approaches
Schill et al., 2019 [39]	Transportation to cultural activities outside urban centres such as medicine picking The importance of urban organizations (such as Aboriginal Friendship Centres) in developing social support networks The role of discrimination and racism Inequitable care as barriers to accessing services in urban centres	Not the focus of this study
Smylie et al., 2011 [41]	Poverty Discrimination Doctor not available in my area Nurse not available Lack of trust in the health care provider Waiting list too long Unable to organize transportation Difficult to get traditional care Health care not covered by Non-Insured Health Benefits (NIHB) Prior approval for services under NIHB was denied Could not afford childcare costs Felt health care provided was not culturally appropriate	Access to traditional medicine
Syme et al., 2011 [40]	Three intersecting issues that impact access to Methadone Maintenance Treatment Stigma and prejudice Social and structural constraints influencing enactment of peoples’ agency Homelessness	Not the focus of this study
Tang et al., 2015 [42]	Racism Discrimination Long waiting times	Not the focus of this study
Tungasuvvingat Inuit, 2017 [43]	No traditional medicine Cannot understand what the health provider was saying Not comfortable with health provider Costs Cannot afford transportation Health services not available after hours	Not the focus of this study
Van Herk et al., 2012 [44]	Not the focus of this study	Safe spaces and safe relational places Belonging and community Engaging the five senses Service provider attitude and commitment
Well Living House, 2016 [45]	Stigma and discrimination Poverty	Not the focus of this study
Wright et al., 2019 [47]	Unwelcoming clinics Poverty	Mothers described four organizational policies that influenced their experiences of using primary care for their infants, including: (a) flexible appointments (b) alternative options for care (c) welcoming receptionists (d) welcoming spaces (e) multi-service clinics
Wylie et al., 2019 [48]	Unwelcoming environment Stereotyping and stigma Practice informed by racism	Not the focus of this study

## Data Availability

No new data were created or analyzed in this study. Data sharing is not applicable to this article.

## Supplementary Materials

The following supporting information can be downloaded at: https://www.mdpi.com/article/10.3390/ijerph20115956/s1, Table S1: Barriers and facilitators of accessing health care among First Nation, Métis and Inuit peoples in urban areas of Canada.
