# Supplementary material for: First Nations, Inuit and Métis Peoples Living in Urban Areas of Canada and Their Access to Healthcare: A Systematic Review
Source: Int J Environ Res Public Health. 2023 May 25;20(11):5956. doi: 10.3390/ijerph20115956 (PMC10252616; doi:10.3390/ijerph20115956)
Supplement: Supplementary file 1 [file ijerph-20-05956-s001.zip › ijerph-2343852-Supplementary/ijerph-2343852-Supplementary Table S1.Barriers and facilitators.pdf]

Supplementary Table S1. Barriers and facilitators of accessing health care among First Nation, Métis and Inuit peoples in urban areas of Canada.

Author, year	Aims / objectives	Barriers to accessing health care	Facilitators to accessing health care
Aboriginal Health Access Centres, 2015 [14]	1. To provide comprehensive health and social services to Aboriginal peoples across the province	**Main themes:**<br>Not applicable (N/A)	**Main themes:** Wholistic wellbeing, culture as treatment, Indigenous community led and run primary health services<br>• AHACs approach health and wellbeing in a wholistic way, providing not only clinical services, but also community designed teachings, languages, medicines and ceremonies<br>• AHACs are community health spaces that offer a sense of belonging<br>• As spaces of respect, truth, humility, honesty, courage, love and wisdom<br>• 50% of patients are people without insurance<br>• *"...mix of non-native and Anisinaabe ways, respectful of both workers..traditional healing is done really well"*
Aboriginal Health Access Centres, 2016 [13]	1. To provide comprehensive health and social services to Aboriginal peoples across the province	**Main themes:**<br>N/A	**Main themes:** Wholistic wellbeing, culture as treatment, Indigenous community led and run primary health services<br>• The Sector provides health services in the following Indigenous languages: Oji-Cree, Cree, lnuktitut, Iroquois, Mohawk and Ojibway<br>• Most AHACs provide home and community visits for primary care. The clients that they typically see are complex: frail seniors, those requiring palliative/end of life care, those with mental health problems, disabilities and isolated/remote<br>• The sector also provides primary care services through Ontario Telemedicine Network (OTN)<br>• Traditional healers, counsellors, medicine people, Elders and traditional teachers work with people on traditional healing services
Auger et al., 2016 [36]	1. How does participation in health circles, based on traditional Indigenous knowledge, impact the	**Main themes:** Racism, discrimination, mistrust.<br>• Frustrations trying to get traditional healthcare - "*there is not enough out there/not aligned with the system*". "It's always easier for me to go to traditional first –	**Main themes:** High value of traditional healing that is based on relationships. Increase in owning their own health and knowing about other options for health.

	health of Indigenous peoples?<br>2. Will incorporating traditional and holistic approaches increase access and utilization of health services?	because that connection seems to be right away. If I go to my doctor I don't have that relationships and I go to her when I'm sick. Yet with the medicine man, many of them, I have close relationships with."<br>• "*Doctors today don't know who we are, especially when we are using walk-in clinics. Our traditional doctors knew us, they knew our family, and they talked to our ancestors in ceremony. If we got sick, our parents knew where to go, and not just to one person, there were different people in the community.*"<br>• Some participants identified barriers to accessing traditional health care, including the mainstream rhetoric, which often denies the value or effectiveness of traditional health care. Participants spoke to areas of health human resources, citing the need for mandatory cultural competency training for all staff in health care, culturally appropriate health services and programs (e.g. more Indigenous-run treatment centres and supports for residential school survivors) and the need to make mental health and substance use a priority.	• Circles were amazing but need more opportunities to practice culture as healing (e.g. more venues, more programs).<br>• Participants made more of an effort to seek out a traditional healer in the community to learn more about medicines and plants.<br>All participants felt more ownership over their health and health care choices, and demonstrated an increased ability to advocate for their health needs; e.g. "*I feel like more of an advocate for myself now that I know what I want*"<br>• Participants identified an increased understanding of different health care options, both western and traditional, and a willingness to explore these (e.g., naturopaths, acupuncturists). One participant said, "I've become open to all forms of wellness. I have an increased willingness to try more forms of health and wellness from attending the workshops."
Auger, 2019 [25]	1. To explore and understand Metis people's experiences with mental health	**Main themes:** a need for increased access to culturally responsive health care spanning both Western and traditional systems.<br>• Others emphasized that they have found traditional practices to be more effective for supporting their mental health, through addressing root causes:<br>• 'I've gone to healing circles and stuff and I find them way more helpful than some doctors… they just want to fix the problem that's right in front of them but usually that leads to more problems'.<br>• lack of culturally safe mental health practitioners, and historical trauma rooted within health-care experiences:<br>• 'My grandmother was hospitalized, my Metis grandmother. She was given electric shock. She was so heavily medicated. I was terrified of medication all my life because of seeing her so heavily medicated' (Participant 14).<br>• Participants also spoke about the danger of disclosing<br>• spiritual gifts to Western health-care providers, as they may be mis-labelled through clinical diagnosis.	**Main themes:** wellness was understood as a whole, where mental, emotional, spiritual and physical health.<br>• Participants spoke about the importance of community connectedness and cultural continuity, as one participant noted, "to have wellness, it means having access to your culture and to resources and support, and not having to do it alone"<br>the importance of culturally safe care and funding for long-term counselling and trauma supports:<br>• "I also feel that there are Metis people in British Columbia that could use definitely more resources, more supports in terms of accessing mental health services, access to counselling, access to trauma counselling, access to trauma treatment, [and] access to extended health supports…"<br>• Many people spoke also about the importance of both<br>• Western and traditional healing:<br>• 'I do see a clinical counsellor every couple of weeks but I don't see that as being more helpful than going to

			the beading group, than going to Metis Night at the Friendship Centre'<br>• Participants spoke about the ways in which their own journeys with mental health have impacted their passion for helping others. Participants spoke about the intergenerational nature of their journeys with mental health and wellness; some noted they are healing for themselves and for their ancestors
Barnabe et al, 2017 [46]	1. Main outcome measures: Accessibility, acceptability, effectiveness, and cultural safety were evaluated as measures of quality for the model of care	**Main themes:** Long waiting periods, transportation, language,<br>• difficulty getting the primary care physician to make a referral (n = 9)<br>• difficulty getting an appointment with the non–family physician specialist once referred (n = 4)<br>• having a lack of non–family physician specialists in the geographic area (n = 5)<br>• having to wait too long to see the non–family physician specialist from the time of referral (n = 6)<br>• appointments being canceled or deferred by the physician's office (n = 2)<br>• Once a non–family physician specialist appointment was secured, participants reported having to wait too long in the specialist's office on the day of the consultation (n = 2)<br>• transportation problems (n = 4)<br>• language problems (n = 1)<br>• 1 participant decided not to attend owing to fear of diagnosis and procedures at the visit	**Main themes:** No discrimination, easy to access the service, good communication with patients<br>• Nearly all participants (95%) disagreed (21%) or strongly disagreed (74%) that they felt discriminated against because of race or ethnicity<br>• They did not perceive that assumptions were made about their level of education or income, or that less attention was paid to them based on race or ethnicity (24% disagreed and 68% strongly disagreed)<br><br>At the Elbow River Healing Lodge clinic specifically:<br>• 55% (21 of 38) believed it would be easy or very easy to access arthritis specialist services at the clinic, but that percentage increased to 87% (33 of 38) after their experience with the model of care<br>• Overall 89% (34 of 38) reported they were satisfied (11%) or very satisfied (79%) with the arthritis model of care<br>• Coordination of services by health care professionals in the clinic was highly rated (63% excellent and 26% very good).<br>• Measures of communication acceptability included patients believing that their health concerns were taken very seriously (79% strongly agreed and 18% agreed) and results of the examination and investigations being explained to all participants<br>• Most participants perceived being supported and encouraged by the physician (66% strongly agreed and 26% agreed)<br>• A shared decision-making approach was perceived by most, with 84% (32 of 38) reporting that a treatment

			plan was worked out in collaboration with the physician<br>• Most also reported perceiving respect (66% strongly agreed and 24% agreed) and being treated as an equal (63% strongly agreed and 26% agreed)
Beckett et al., 2018 [49]	1. to explore the relationship between different health access barriers and diabetes among an urban First Nations group living in Hamilton, Ontario, Canada, while adjusting for covariates, including age, sex, body mass index (BMI), physical activity levels, alcohol consumption and smoking status	**Main themes:** Not having NIHB insurance, long waiting lists, income, income, poverty, not culturally appropriate.<br>A statistically significant relationship between self-reported diabetes and:<br>• not being covered by non-insured health benefits (NIHB) (OR: 3.66, 95% CIs: 1.32 to 10.15)<br>• prior approval for coverage under NIHB being denied (OR 3.48, 95% CIs: 1.36 to 8.87).<br><br>A statistically significant relationship between self-reported diabetes and:<br>• the waiting list to access health services being too long (OR: 2.00, 95% CIs: 1.19 to 3.38),<br>• not being covered by NIHB (OR: 3.66, 95% CIs: 2.00 to 6.70),<br>• prior approval for coverage under NIHB being denied (OR: 3.47, 95% CIs: 1.81 to 6.67)<br>• income (OR: 1.11, 95% CIs: 1.03 to 1.19)<br><br>A statistically significant relationship was found between self-reported diabetes and:<br>• feeling that healthcare provided was inadequate (OR 8.54, 95% CIs: 1.40 to 53.40)<br>• respondents feeling healthcare provided was not culturally appropriate (OR: 17.87, 95% CIs: 2.90 to 110.24)<br>• income (OR: 1.29, 95% CIs: 1.03 to 1.63)<br>• Using the weighted generalised linear mixed methods model approach, feeling that health services were not culturally appropriate was significantly associated with self-reported diabetes (OR: 12.70, 95% CIs: 2.52 to 57.91).	**Main themes:**<br>Not the focus of the study.

Benoit et al., 2003 [51]	1. Our research focused on asking Aboriginal women living in Vancouver's Downtown Eastside (DTES) whether the service delivery model employed at the Vancouver Native Health Society (VNHS) provided them with the appropriate professional services and educational programs needed to take control of their health and healing.	**Main themes:** Difficult communication, poverty, no traditional healers.<br>• Vancouver Native Health Society staff commented "*Are we doing enough? I don't think so. I just don't believe that I should have a family that comes in to me here on a Friday afternoon saying, 'we've had no food in the house for the last four days'*, or that *'I haven't been able to feed my baby for the last couple of days'*.<br>• Aboriginal woman participant = "*Aboriginal women in the DTES Even though I'm a strong woman on the outside, deep down I'm hurting and many of us women are like that. But we don't show it because we don't want people to look at us like we're less than we are. It's just our way; we walk with our head high and proud.*"<br>• clients and staff said "*If you're going to do something for Native people, I think you need to be like traditional and spiritual 'cause that's what Native people are about, you know? Do they have elders in the* Vancouver Native Health Society*? Do they have Native healers? No, they have doctors! Gee, that's not my system, that's not where I come from. You know, for a First Nations organization, how come there's like, not a lot of Natives working? It's like the same White system with just a different name on it.*"<br>• Vancouver Native Health Society staff said "*They're difficult people, most of the people we see, could quite easily get rejected in a standard practice, you know, because of difficult behaviour. They're impatient, can't wait very long, they can be aggressive, they don't dress normally, don't necessarily take baths, they can't make appointments and [often don't] follow up. So you have to try to be more tolerant of this sort or behaviour.*"	**Main themes:** Good communication, wholistic wellbeing approach, Indigenous health services.<br>• Participant said "*I went to another downtown clinic and the doctor that I had was giving me constantly the same pills all the time when I was getting sick. I went over to the Native Health and the doctor there, as soon as she saw me, said, 'Get to the hospital.' And now she is my doctor. She is somebody who cares and takes the time to listen to me. Where, down at my other doctor, it was in and out and bye*".<br>• Participant said "*I prefer to be around First Nations people because they're the ones who understand where we come from. When you go in there [the Clinic], a non-Native person will look at you as a client. But a First Nation's person will look at you like a friend, but will maintain her professionalism*".<br>• Sheway staff member said "*Sheway I think the unique feature of Sheway [is that] it helps to make health and social services more available and more accessible and more relevant to women, as well as being a safe place.*<br>• Sheway client said "*Sheway's mandate is that when you have a baby that's eighteen months, you're done your time here. Sheway says it's time to move on and that's when you need the most help, more support, more understanding. That's when you need the next step*".<br>• Sheway staff member said "*We spend a lot of time talking about birth control with a lot of women. I know the nurses will counsel on safe sex; we all do. If we know that a woman is practicing unsafe sex we are able to discuss [condom use] as a form of prevention. We talk about prevention in regard to safetiness for the women working in the sex trade. We talk about looking out for themselves, making sure they have a date list*".

Benoit et al., 2019 [50]	1. Describe stressful life experiences?	**Main themes:** Racism, Sixties Scoop, the Indian Act, and Indian Residential Schools, inadequate health services, socio-economic insecurity, negative stereotypes and judgements, and a lack of recognition of Indigenous knowledge systems.<br>• Past events were directly linked to colonial activities and policies such as the Sixties Scoop, the Indian Act, and Indian Residential Schools<br>• Current events related to the inherited outcomes of colonialism that focused on inadequate health services, socio-economic insecurity, negative stereotypes and judgements, and a lack of recognition of Indigenous knowledge systems.<br>• *"I was at the doctor's office, I dislocated my arm, and they kept putting my chart down, and my son was waiting for me at home, at his place, and it was like, oh my god, my son is waiting for me, why are these people putting my chart down to the bottom? I've been waiting like everybody else, how come they keep putting my chart down? That pissed me off. I put my arm back into place and I walked out. That's crazy, I shouldn't have had to do that"*	**Main themes:** Not the focus of the study
Browne et al., 2011 [52]	1. To examine the experiences of access to primary care services from the perspective of Aboriginal people seeking care at an emergency department	**Main themes:** Discrimination, difficult communication with staff.<br>• Patients were concerned with how health care providers might be responding to them on the basis of their appearance as people living in poverty, as residents of the inner-city neighborhood, or as people affected by substance use and addictions. Patients' concerns centered on the potential dismissal or diminishment of their health issues because of how they would be "read" by providers. One 48-year-old Métis woman described it in this way:<br>• *'Well it seems like when my husband and I [were renting] in a well-to-do neighborhood . . . you're accepted as being normal class, everyday citizens. But as soon as you're known to have come from the [inner city], the first thing that's thought about you is that*	**Main themes:** Caring about the person, wholistic wellbeing approach<br>• Patients described how significant it was when providers in the ED conveyed concern about their general well-being, or acknowledged their presence in some way; for example, when they were asked if they wanted a drink or a snack; when they felt listened to attentively for a few moments; or when providers stopped very briefly to ask how they were feeling as they were waiting to be seen

		you're a drug addict or a drunk. . . . Like as soon as I tell them that I did interferon treatment for hepatitis C, the first thing they ask me is, when was the last time I used.'<br>• "They have attitude, especially the admitting clerks. . . . I don't know, maybe because I'm a drug addict . . . maybe because I'm Native. . . . Today they're nice. . . . But I avoid coming here in a big way." Some people's need to be able to walk to the closest ED (having no money for a bus or taxi).	
Cameron et al., 2014 [53]	1. To investigate and address inequities in access to health care services for Aboriginal peoples and inner-city residents in partnership with Aboriginal people, health care professionals, and university researchers<br>2. To investigate Aboriginal peoples' experiences of access to health care services in the urban, rural, and inner-city settings	**Main themes:** limited access to specialized care, long waiting times, barriers in the communication and understanding of medical jargon, and barriers in the interaction with health care professionals.<br>• the fear of judgment by mainstream health care professionals affected the quality of interactions at the individual level<br>• emergency staff at times did not take the time to further assess participants' needs and concerns or explain health conditions, test results, or even estimated waiting times<br>• one element that affects the health of Aboriginal peoples is the tension and distrust that has historically marked the relationship between Aboriginal people and the health care system<br>• when care expectations were not met, participants felt powerless and bewildered. Their trust toward health care professionals and health services diminished rapidly<br>• *'Last time when I was here I couldn't understand what the staff was saying to me because I was so sick. No one was there to translate for me if I wanted painkillers or even to phone my family for me.'*	**Main themes:** Good communication with health professionals, having a family member go with you.<br>• When there was understanding of what was happening, there was satisfaction and recognition of the good work of health care professionals<br>• This time it was not as bad because my daughter came with me as an escort. I felt I was treated alright. She stayed with me till I was moved to the unit. I felt this time around the staff treated me good<br>• *'This time I understand as the doctor talked slow to me and when I don't understand the question I asked him to explain to me better. I feel more comfortable now. I feel a little ease this time.'*
Carter et al., 2014 [15]	1. To explore how women living with HIV understand, experience and negotiate 'place' and 'space' in attempting to access HIV-related care in British Columbia	**Main themes:** Racism, discrimination.<br>• *"I hate going to our hospital ER... If you're Aboriginal and you're complaining. Say if you had been drinking that night or whatever, it does not matter. They are... all White, all the staff, and they're very racist...They will help you eventually. But you are so tired at one point that you're either trying to curl up on a chair or you've got to leave. And then you haven't been helped and they're saying well you know you were next in line. Well*	**Main themes:**<br>Not the focus of the study.

		*that's bullshit. "*You know exactly where you stand in line and it's right at the very bottom of the list..."	
Denison et al., 2014 [16]	1. What are Aboriginal women's experiences of accessing healthcare services when child apprehension is a threat<br>2. How can the quality of healthcare delivery to Aboriginal women and children be improved when child apprehension is a threat?	**Main themes:** Racism, discrimination.<br>• One mother said…[L]ike at [hospital name] I had pneumonia and I was really, really sick…and they discharged me I wasn't even better yet. My pneumonia hadn't even [gone away] and it was during wintertime. And…one of the nurses came in and said that the doctor is discharging you. I said I'm not even better yet and she said, well it's time for you to go now, you need to get your stuff and you need to go, don't let me call security. And sure enough she called security. Security literally came in, grabbed me behind my arms, dragged me down the hallways and threw me out the door, with pneumonia, in wintertime. And I went back in I said can I at least get a bus pass, a bus ticket? And they said this is not a charity this is a hospital. And right now I'm almost in tears… (WP9)<br>• The women believed that they were discriminated against for being Aboriginal, appearing poor, for substance use, or for being teenage mothers. Consequently, racism, discrimination, judgment and the fear of child apprehension had an impact on the women's decisions and experiences with health care in two main ways: it deterred the women from visiting their children while in hospital and/or it deterred the women from seeking healthcare services for themselves.	**Main themes:**<br>Not the focus of the study.
Environics Institute, 2010 [17]	1. Is having access to traditional healing practices more important, less important or equally important to you as access to mainstream healthcare services?	**Main themes:**<br>Not the focus of the study.	**Main themes:** Traditional healing, easy access to services.<br>• Almost half (45%) of urban Aboriginal peoples say access to traditional healing practices are equally important to them as access to non-Aboriginal or mainstream health care services<br>• The view that access to traditional healing practices is more important than access to mainstream health care services is more evident among Inuit (37%) and status First Nations peoples (36%) than among non-status First Nations peoples (25%) and Métis (20%)

			• Status First Nations peoples (37%) are more likely than Métis (24%), non-status First Nations peoples (18%) and Inuit (13%) to say it is *very* easy for them to access traditional healing practices<br>• Access to traditional healing is also judged considerably easier by urban Aboriginal peoples living in Toronto (52% very easy). However, the ease or difficulty of access does not vary significantly by personal health status
Firestone et al., 2014 [19]	1. To work in partnership with Aboriginal stakeholders to generate a culturally relevant, representative baseline health data set for three urban Aboriginal communities in Ontario, Canada	**Main themes:** Transportation, doctor not being available; income, poverty, services not covered by Non-Insured Health Benefits and lack of trust in healthcare provider<br>• Among First Nations adults (n=554)<br>o 16.7% felt that availability of health services in their community was excellent<br>o 43.3% felt it was good<br>o 28.9% felt it was fair<br>o 11.1% felt it was poor<br>• 40% of the population felt their level of access to healthcare was fair or poor, despite the geographic proximity to extensive health and social services that the City of Hamilton provides<br>• This substantiates the idea that just because the services are geographically proximate does not mean that they are accessible to First Nations people<br>• 47.9% of First Nations adults living in Hamilton reported that long waiting lists to see a specialist were a barrier	**Main themes:**<br>Not the focus of the study.
Firestone et al., 2015 [18]	1. To work in partnership with Aboriginal stakeholders to generate a culturally relevant, representative baseline health data set for three urban Aboriginal communities in Ontario, Canada	**Main themes:** Discrimination.<br>• 41.8% experienced discrimination which prevented or delayed receiving health care	**Main themes:**<br>Not the focus of the study.

Goodman et al, 2017 [20]	1. To explore how multiple forms of discrimination and oppression shape the healthcare experiences of Aboriginal peoples living in a marginalized community	**Main themes:** Participants' experienced medical dismissal often which resulted in disengagement from care or delay in care.<br>• Individuals drew upon a seemingly collective narrative about how others before them have experienced adverse care, which informed personal understandings and experiences of healthcare.<br>• Participants shared numerous examples of being threatened by hospital security or dismissed by staff. One woman described an encounter where her attempt at social niceties resulted in her involuntary discharge from the hospital<br>• All participants repeatedly referenced feelings of being "treated differently" in their healthcare interactions. 'So [the nurse] showed me how to [inject], but she was so mean about it. She was not accommodating. She said I should know how to do it myself. They treated me like crap and I know it was because I was Native. We all know because of the look - there's a look. When you need the medical care we put up with it. We shouldn't have to.<br>• 'I was doubled over in pain. [The doctor] asked for a scale of 1–8 [for pain]. [Participant replied] 8. […] A Slavic man and nurse came to assist me. I noticed his accent. I [dated] a Slavic guy. Asked where he was from? [He answered] 'None of your business!' and I said, 'What's the problem? Is it my skin colour or my postal code?' He kicked me out. [Nurse responded], 'You're outta here!' I've never been kicked out of anything except a bar. [Female participant #1, Talking circle #1]'<br>• subliminal forms of institutional racism were also made evident. One woman spoke to the detriments faced by many Aboriginal women (e.g., poverty, substance use), while at the same time alluding to the negative impacts of cultural disintegration. She continued on to express how hospital policies exacerbate cultural barriers to health and wellness for Aboriginal peoples<br>• '*I lie to my doctor about drugs I use. They have no idea what I'm on, but because of the area [DTES] I'm in he*	**Main themes:** Good communication with health professionals, wholistic wellbeing approaches.<br>• A participant recalled positive interactions with a physician that he perceived was competent in addictions medicine. Thus, having one's needs understood promoted a sense of equitable care.<br>• '*I love Dr. [anonymous]. He knows addiction, but I'm really finding we need an addictions specialist. We really don't have one. Apparently they have at [name of a clinic], but I can't go because [name of a clinic] is my clinic. They tell me I can't, but I wonder is it because I live in the DTES? Or because I'm Native? Or [emergency medical services] tells them not to take me? 'Get the fuck out of my office', doctor told me when I needed meds to stop seizure. So first thing, I went to the liquor store so I wouldn't have a seizure. I saw an aura. Doctor didn't put it together. I don't think there are enough people qualified in this country on addictions. It's sad to say.'*

		doesn't want to give me as much and wants to lower my dose. I have Crohn's Disease and cocaine takes care of it so I sell medication too'	
Goodman et al., 2019 [21]	1. To examine the relationship between social support and health within the urban context, based on findings from a collaborative research project with an Indigenous-led youth program located in Winnipeg, Canada	**Main themes:** residential mobility and racism negatively influenced the types of social support and relationships formed, and called for improved access to health-promoting social programs.<br>• "If you are constantly moving, it's hard to put a lot of effort into building new relationships…it is hard to find positive supports in your life"	**Main themes:** Culturally based services.<br>• When asked to depict a 'healthy urban community', the youths' photographs focused on public spaces as opportunities for positive social interaction: '*I was taking pictures of community centres, basically for interaction of young youth or older people, drop-in-centres to come in and get to know our young youth and to be involved in each other's lives and be part of the community…I took a picture of a park. Basically, for families and friends or whatever to be able to communicate in a healthy way by going to a park or going somewhere for activities or meeting people.'*<br>• many also associated the concept of stability to culture and lifestyle. One shared a photograph of a pow wow to epict what she felt was important to her health: 'It's a pow-wow. It's good to have tradition and stability in your life. Well, the way I look at it just like, it's a way of life, it's stable, secure. It's a lot better than doing drugs and stuff. It's a way of living.'<br>• '*You know what I think? There needs to be a program out there that works with the family instead of just the person who is in the gang because that is where it is going to go back to the home environment, like if there is drinking going on, or if there is people who are selling drugs, like they are going to influence the individual to join and so I think a whole like a holistic approach to fix the family so the family can live in a healthy environment*.'
Health Council of Canada, 2003 [22]	1. To learning about Aboriginal maternal and child health realities "on the ground", including practices supported by governments and communities that could be considered "promising' in	**Main themes:**<br>Not the focus of the study.	**Main themes:** Traditional healing, Indigenous community-based services.<br>• Traditional knowledge and cultural approaches: Revitalization and incorporation of traditional knowledge, culture, or use of languages in maternal and child health programs and services. This includes programs that address "wellness" as opposed to

	addressing the discrepancies in health status. Par		"illness" and are holistic (emotional, spiritual, physical, mental, intellectual) approaches.<br>• Community-based and community-focused approaches: Programs, services, and approaches that are developed at the community level, or large-scale federal, provincial, territorial, or regional programs that are adapted at the community level. 3. Collaboration and integration: Bringing together, working together, combining funding—or other collaborative approaches between Aboriginal maternal and child health programs and services, or linking with other community programs and services (housing, employment, social services).<br>• Training and human resources: Successful ways of recruiting, training, and retaining Aboriginal people who work with and support First Nations, Inuit, Métis mothers and children. This topic can also include successful ways that non-Aboriginal organizations and health practitioners are trained, recruited, or work in Aboriginal maternal and child health (cultural competency, cultural safety).<br>• Policy and funding: Broad policies or funding programs at the federal, provincial, territorial, or Aboriginal government level that have improved Aboriginal maternal and child health; something that could be seen as a model or promising approach
Health Council of Canada, 2012 [24]	1. To improve Canadians' understanding of the issues that underlie disparities in health status and in access to health care between First Nations, Inuit, and Métis people and the larger Canadian population<br>2. To identify practices that are improving health status (in the broad sense of health, wellness, and community healing) and	**Main themes:** Not the focus of the study.	**Main themes:** welcoming, feeling culturally safe, feeling like you belong.<br>• The physical and emotional environments feel familiar, welcoming, warm, and comfortable<br>• To feel respected as equals and heard when accessing services<br>• they can trust service providers and the providers are interested in them as people<br>• Clients feel safe and comfortable, and providers feel that they understand the impacts of community or culture on their clients' health and wellness<br>• Culturally safe health care services offer clients on-site access to both traditional holistic approaches and Western approaches to care

	access to health care for First Nations, Inuit, and Métis people		• people may more easily feel that they belong and are respected, and that their values and practices are recognized and reflected<br>• when they access services at or through organizations that are operated by or under the control of First Nations, Inuit, and Métis people<br>• For non-Aboriginal people working with Aboriginal people, "it should not always be about what [they] can do for First Nations, Inuit, or Métis people." In a culturally safe environment, non-Aboriginal people recognize and acknowledge that they too will<br>• learn and gain competency from their relationships with Aboriginal people<br>• Health professionals advocate for equitable and culturally safe care for the First Nations, Inuit, and Métis people they serve
Health Council of Canada, 2013 [23]	1. To learn more about the health challenges of older Aboriginal people, and the ways in which Aboriginal communities, health care providers, and governments are working to improve health care services for First Nations, Inuit, and Métis seniors	**Main themes:** Lack of nurses and retaining nurses,	**Main themes:** Integrating culture into care/participating in traditional activities, cultural safety, hiring local, self-governance, community aides to deliver services, language, personal connection to the local community<br>• From an elder care perspective, the aides can spend more time with elders than the nurses do; also, they have those personal connections and speak the language
Heaman et al., 2015 [27]	1. To explore the perceptions of women living in inner-city Winnipeg, Canada, about barriers, facilitators, and motivators related to their use of prenatal care	**Main themes:** transportation and child care, could not afford transportation to get to prenatal appointments, long periods in a waiting room, negative personality characteristics of care providers as barriers to PNC, such as being rude or abrasive, distracted, or not caring.<br>• "*I had no idea where to go. I was terrified about, like, there is [sic] so many doctors out there. I didn't know which one to go to, so I didn't even go*" (G1P0, 18 years)<br>• The receptionist said, "Okay, we will see you at 11 weeks. When will that be?" And I remember thinking, "What? You're going to see me at 11 weeks! I am 5 weeks pregnant now. That's 6 weeks until then."	**Main themes:** transportation assistance, convenient location of services, positive care provider qualities, and tangible rewards. Women were motivated to attend prenatal care to gain knowledge and skills and to have a healthy baby.<br>• '*Dr. [name of obstetrician] tells me the baby is okay, she tells me she is growing fine, and the heartbeat is looking good and all that, and she just tells me what is going on. I like that about her.*'<br>• Some of the terms used to describe these characteristics were *nice, kind, reassuring, caring, thorough, patient*, and *knowledgeable*.<br>• "*He just took the time. He respected the questions that you had, and he was a lot more thorough, it seemed,*

		• The doctor himself is so abrasive—Flies in to the room, does what he needs to do … it doesn't really seem like he cares, and he is out the door and on to the next patient. … I feel so rushed that I don't actually get to talk about things that are pertinent to my pregnancy. And so I leave the office feeling unsatisfied or not voicing concerns<br>• Some women commented specifically on the shortage of midwives and finding "they were all booked up," whereas others noted the shortage of obstetricians in the city<br>• The lack of consistency of PNC providers and subsequently receiving impersonal care was an issue for women who received care from medical residents in teaching clinics. One woman stated, *'Like you don't even see the real doctor unless they [residents] think that it is necessary for you to have to. And then it is a different doctor all the time ... they don't know you or what is going on.'*	*and attentive ... He ... uses humor and is a very positive person"*<br>• "*They [midwives] cared more about the experiences I was having ... they cared more about the whole entire situation*"<br>• Family members or friends encouraged them to attend PNC, played a key role in helping them find prenatal services, and attended PNC sessions with them or provided child care so that they could attend PNC visits<br>• Some women commented that PNC attendance was facilitated by the availability of bus service and convenient bus routes, having a car, or receiving bus tickets to attend a Healthy Baby community support program.<br>• You got information; it was free; you got to meet other people that were going through the same stuff as you. Learn stuff that you may have forgotten, got a little snack on the side. And they give you the recipe on how to make it and some of the ingredients all for a dollar … You got everything you basically needed in one spot.
Heaman, 2018 [26]	1. to explore perspectives of women and health care providers about PIIPC	**Main themes:**<br>• Themes included better understanding of other programs arising from involvement in PIIPC, improved communication, benefits of team work, and positive changes in service delivery (e.g. more accessible, convenient).	**Main themes: convenient, good communication.**<br>• Women described access to PC as convenient and coordinated; appreciated flexible scheduling and receiving incentives and assistance with transportation; and commented on positive relationships with providers, using descriptors such as helpful, respectful, and nonjudgmental.<br>• The PIIPC project reduced barriers to care and facilitated communication between providers and programs, resulting in improved use of PC by inner-city women.
Hole et al, 2015 [28]	1. to interrogate practices within one hospital to see whether and how CS for Aboriginal patients could be improved	**Main themes:** structural violence that reproduces experiences of institutional trauma in hospital.<br>• the social and historical context within which health care practices are delivered<br>• *'You can hear them [nurses]. And, you're in [the] emergency room and they're talking real nice to*	**Main themes:** Positive culturally safe experiences, were described as interpersonal interactions with feelings, being visible, being heard, being respected, treatment as a "human being".

		someone who's not a Native and then they come in to me and their attitude changes, eh? So, they're not all like that, but I run into that quite a bit.'<br>• Participants acknowledged an under-resourced health system; the rules, policies, and intervention priorities of that system; and the physical space of the hospital and wards.<br>• Participants described how various hospital practices reflected a particular culture of family that lead to experiences of frustration and loss.<br>• Some of their rules I think are stupid. I don't think they're effective. Like the whole two people in a room rule. They don't get the fact that this is so significant for us. They have their idea of what is medically safe. And it's more that they're able to get to the patient and work with the patient than it is for the patient to have family support. We managed that. When they needed to come in and deal with grandma then we left.<br>• Interpersonal interactions with health care providers and hospital staff were a third factor contributing to Aboriginal people's negative health care experiences. Many participants described situations when they were not listened to, when they were not believed, and/or when they were judged in a negative light.	• "They respected him. They were, any little thing that he had happening to him, they were on it right away when he was in the hospital."<br>• When my mom passed on I thought it was just great that, uh, staff members had let us do smudging in the room she was in [and] that we didn't have to go some place else. And they respected what we wanted to do.<br>• When I'm ready to go home, they ask me if I'm feeling comfortable about going home again and I tell them, 'yeah'. And then they ask me all the things, "Is there going to be somebody there to, to help you and my family? Is there . . ." They'll look after me for a week . . . And they're on top of everything.<br>• unexpected gestures of kindness
Kitching et al, 2020 [29]	1. to assess the association between experience of discrimination by healthcare providers and having unmet health needs within the Indigenous population of Toronto	**Main themes**: Racism and discrimination.<br>• prevalence of discrimination by a healthcare provider was 28.5%<br>• prevalence of unmet health needs was 27.3%<br>• Discrimination by a healthcare provider was positively associated with unmet health needs (OR 3.1<br>• 36% did not have access to a regular healthcare provider	**Main themes:**<br>Not the focus of the study.
Lawrence et al., 2016 [30]	1. to assess whether there were associations between oral health- related outcomes and self-reported racism	**Main themes:** Racism, discrimination.<br>• racism experienced by Aboriginal women can be a barrier to accessing dental services<br>• A third of women experienced racism in the past year<br>• Outcomes significantly associated with incidents of racism included:	**Main themes:**<br>Not the focus of the study.

	2. to examine associations between oral health-related outcomes and self-reported racism persisted after adjusting for significant covariates in our sample.<br>3. to compare the prevalence of self- reported racism among the three countries collaborating on an early childhood caries preventive trial<br>4. to compare the findings with prevalence estimates reported in First Nations-governed, national health surveys in Canada	○ wearing dentures<br>○ off- reserve dental care<br>○ asked to pay for dental services<br>○ perceived need for preventive care<br>○ flossing more than once daily<br>○ having fewer than 21 natural teeth<br>○ fear of going to dentist, never received orthodontic treatment<br>○ perceived impact of oral conditions on quality of life	
Loyola-Sanchez et al., 2020 [31]	1. To explore patient preferences that influence decision-making in the management of rheumatoid Arthritis (RA) by indigenous patients living in southern Alberta, Canada	**Main themes:** good relationships with health providers,<br>• *Clinical factors*. Relationship with health care providers. The characteristics of the relationship that patients had with their health care providers were essential for the acceptance of pharmacologic treatment. Relationships based on trust resulted in greater acceptance of medications.<br>• "…for native people there's a lot of guard up because there's hatred to both, whether who we can see can take care of us because I believe that there's a way people look at First Nation people as not as smart as they should be, so for us to have that trust,…we need to look at you in the eye and trust you…"	**Main themes:** Increasing patient–provider trust. Patients' narratives identified that patient–provider trust could be fostered by an environment that is safe, collaborative, and professional.<br>• A safe environment refers to a space where patients can find empathy, a holistic approach to health issues, respect for their knowledge and experience, and acknowledgment of cultural differences<br>• A collaborative environment requires a mutual understanding between patient and providers to be actively involved in decision-making<br>• A professional environment requires health care providers to be perceived as knowledgeable, experienced, and reliable, exhibiting honesty, directness, effective communication, and active listening skills<br>• "I have to trust them (health providers) and well, having access to them when, it's an important thing too (to consider accepting a prescription)" (47-year-old woman)

McCaskill, 2011 [32]	1. To gain a better understanding of the circumstances of Aboriginal people in Toronto to fill a gap in our knowledge and to put forth recommendations as to what better meets their needs	**Main themes:** Racism.<br>• 58% of Aboriginal men considered racism by non-Aboriginal people in Toronto and indicated that it is prevalent in a diversity of systemic and institutional contexts<br>• One respondent shared her experience in the hospital:<br>o *'I've been to the hospital over the years, and they've asked me if I've been drinking with my stomach problems. Meanwhile I've been sober for many years. So there is an assumption'.* (Aboriginal Women's Focus Group)	**Main themes:** Indigenous led and run services.<br>• Aboriginal Women have two main agencies that are there solely for the purpose of supporting them. Anduhyuan Women's Shelter and the Native Women's Resource Centre
Mill et al., 2008 [33]	1. To explore HIV testing and care decisions of Canadian Aboriginal youth.<br>Objectives:<br>1. Why do Aboriginal youth have an HIV test?<br>2. Why do they not have an HIV test?<br>3. What are the testing behaviours of Aboriginal youth and what types of services do they use?<br>4. Among Aboriginal youth, what is the relationship between HIV testing and the decision to initiate treatment?	**Main themes:** Discrimination.<br>• 13% were worried about being discriminated against<br>• 6% it takes too long to get the results<br>• why they did not seek care immediately, interview participants reported: being scared or not wanting to think about their disease; being pre-occupied with drugs or alcohol; not wanting to live; not knowing anything about care<br>• *'For me, when I was diagnosed, the doctor where I'm from had just said, 'Okay, you're HIV-positive'...And that was all there is ... I had to tell him that I'm pregnant. I should be on some meds. I was the one that told him that my baby needs the meds to reduce the risks. It's, like... 'you should phone [provincial AIDS service provider] or somebody, and talk to them.' He did...'*<br>• *[Aboriginal organization] gave me a referral ... But that bounced back and forth between answering machines, trying to make the initial appointment for about 6 months ... I didn't really want to go see him, because I didn't want to know my results or my counts or none of those things.'*	**Main themes**: Indigenous culturally based services, wholistic wellbeing.<br>• For the 40 youth who tested outside their current community or the community where they lived at the time of the HIV test, 10 indicated that they did so because "no one knew me," 9 because "I knew the health worker", 6 because "there was no HIV testing in their community", and 3 because "my family was there to support me."<br>• several expressed support for drop in testing sites that provide flexibility in access to HIV testing services. Sam was able to access HIV testing in conjunction with other services offered by a drop-in centre. '*You could be here to use the food bank. Nobody's really sure. And people are coming, going in and out so much, suppose you duck into one of these doors and get tested, then nobody's the wiser that that's why you were here'.*<br>• *'after [they told me I was positive] there was a bunch of counsellors that came to see me to help me deal with it."*<br>• *'That's going good, the doctor, yeah .... [S]he gives me tons of information. I didn't know nothing about care or anything like that, but since I came to [western Canadian city], I need care.'*
Nelson et al., 2018 [34]	1. To examine barriers to health care access for Indigenous people in light of the ways in which	**Main themes:** lack of quality of care; long wait times, racism and discrimination.<br>• lack of quality of care related to delays in diagnosis of a health problem:	**Main themes:**<br>Not the focus of the study.

	broader structures of colonialism influence Indigenous people in their everyday lives.<br>2. To examine these barriers, in order to highlight the individual-level impacts of these broader structures and how they become embedded in the everyday spaces of health care settings.	o denial of medication, in particular pain medication;<br>o delays in seeing a medical professional or lack of treatment altogether cases of serious neglect.<br>• Delays or mistakes in diagnosis were interpreted with great mistrust, suggesting that participants felt they were being deliberately mistreated or discriminated against<br>• With respect to denial of medication, clients reported that health care providers deny them medication because of concerns about substance misuse within the urban Indigenous population<br>• Sixteen of the 65 participants, both health care providers and Indigenous community members, described experiences of perceived racism or discrimination in health care based on Indigenous status, identity, or appearance<br>• one participant described wanting to find a new doctor because her current family physician "*made a comment against Aboriginal people and I didn't like it"*<br>• Another client described feeling uncomfortable when filling prescriptions for herself or her son: Depends on which pharmacy you go to…. as soon as they see me, they always say, 'okay, how are you going to pay for this?' I find it… labeling [me as a First Nations person]…. and I find them, I'm sure they don't mean to, but they always… make me feel like I'm lower than I am. As a person. Because they always ask, like, 'how are you going to pay for this? You even got money?'<br>• one health services worker said, "*I don't think the system is borderline. I think the system is a racist system*" Clients, on the other hand, accompanied descriptions of racist or discriminatory experiences with comments such as "*I'm sure they don't mean to", or "it doesn't bother me anymore'*<br>• In some cases, instances of perceived racism or discrimination were described as discrimination against an Indigenous-led agency	
Nowgesic et al., 2015 [35]	1. To examine how Indigenous peoples living with HIV construct and understand their	**Main themes:** Accessing antiretroviral therapy within the context of living with a substance use disorder was an overarching theme.	**Main themes:**<br>Not the focus of the study.

	experiences accessing ARV therapy	• Indigenous peoples living with HIV felt they had to choose between living with their active substance use disorder and accessing antiretroviral therapy<br>• They felt misunderstood as a person living with a substance use disorder and often felt coerced into using antiretroviral therapy<br>• participants living with a substance use disorder acknowledged their preoccupation with satisfying their desires<br>• Study participants with a drug use disorder spoke about their physicians denying them ARV therapy<br>• I got a massive scolding from him [doctor]. Yeah. He said, "*If you don't straighten out then I'm not going to give you your ARV therapy*." I've had him say that once before, just because I had a struggling addiction problem. Before it used to be cocaine. And this was a few years back. He told me, "*You got to come back with a clean piss test or I'm not even giving you your meds*."<br>• Participant 2 got upset when his physician scolded him for not adhering to his ARVs because he felt as if he was being judged when his physician spoke to him in a condescending manner.<br>• some medical practices pertaining to the co-administration of methadone maintenance therapy (MMT) and ARV therapy. '*basically, it's like they won't get their methadone unless they take that ARV*.'	
O'Brien et al., 2016 [37]	1. To determine population-based prevalence estimates for key health outcomes for urban Aboriginal adults living in London	**Main themes:** Inability to get or afford transportation, poverty, lack of trust in health care providers.<br>• Of people who accessed emergency care, 31% rated the quality of care as fair or poor<br>• Of people who accessed hospital care, 21% rated the quality of care as fair or poor<br>• 26% have been treated unfairly by health care professionals because of their Indigenous identity<br>• 77% indicated that they had experienced unfair treatment more than once in the past 5 years<br>• 67% of Indigenous adults in London said that experiences of racism from health care professionals	

		prevented, stopped or delayed them from returning to health services<br>• 39% of Indigenous adults in London perceived that their overall access to health care services was poorer compared to the general Canadian population	
Pearce et al., 2019 [38]	2. To put forward pragmatic recommendations based on the stories of Indigenous people living with or treated for HCV, with additional perspectives provided by HCV treatment providers, to inform the development of decolonizing HCV care.	**Main themes:**<br>• First: treatment providers must understand and accept colonization as a determinant of health and wellness among HCV-affected Indigenous people, including ongoing cycles of child apprehension and discrimination within the healthcare system<br>• Second: consistently safe attitudes and actions create trust within HCV treatment provider-patient relationships and open opportunities for engagement into care<br>• Third: treatment providers who identify, build, and strengthen circles of care will have greater success engaging HCV-affected Indigenous people who have used drugs into care<br>○ '*Some people do not come to hospital. They wait and wait and wait until it's too late and then when they do come to the hospital, this is where they die. They do not leave the hospital. That I know for a fact. I've lost so many friends and family with the same story. It continues on a regular basis.'*	**Main themes:** Good communication, respectful relationships, trust, wholistic wellbeing approaches.<br>• Safe attitudes were demonstrated by providers who showed sincere caring/empathy (vs. pity), had a good sense of humor (vs. too serious, tense), acknowledged mistakes (vs. lacking humility), moved on quickly from disagreements and mistakes (vs. unforgiving, resentful), and took interest in getting to know patients (vs. indifferent)<br>• Safe attitudes were also demonstrated by doctors who respected patient autonomy and held non-judgemental, strengths-based perspectives on substance use<br>• HCV-affected Indigenous participants who trusted their doctors, most said they felt consistently respected and accepted regardless of substance use and that their provider consistently supported their wellbeing.<br>• '*I love the treatment (at the clinic). The doctor, she's amazing. I'd like to have her as my doctor till the day I die. She's so understanding and she'll speak to me in ways that I understand. She's so supportive, no matter what the decision is that I do. She'll support it, support you. 'I may not like some of your decisions, but I'll always be there for you', you know? Yeah.'*
Schill et al., 2019 [39]	1. To share insights gleaned from a research partnership with a local, urban-based Indigenous community service organisations, the Ki-Low-Na Friendship Society (KFS), to address the gap in knowledge regarding how urban	**Main themes:** transportation to cultural activities outside urban centres, such as medicine picking, the importance of urban organizations (such as Aboriginal Friendship Centres) in developing social support networks, and the role of discrimination, racism and inequitable care as barriers to accessing services in urban centres.<br>• Culturally unsafe environments caused Elders to be fearful of health-care services and to actively avoid using them. '*Our people are very scared of doctors. [. . .] And sometimes we don't trust the doctors. We don't*	**Main themes:**<br>Not the focus of the study.

	Indigenous Elders in Kelowna, BC, are uniquely impacted by the social determinants of mental wellness.	*trust them because we don't know what they're going to give us. And sometimes that can harm our body. [. . .] That's why when I was smoking and I was coughing for three days, I didn't go to the hospital because I'm scared of hospitals. [. . .] A lot of our people are like that. It's, sometimes it's trust, sometimes we're shy, sometimes we just - that's just the way our people are, you know.'*<br>• 'I *think that I have to mention cultural safety. It's so important. It's something that should be a way of being for everyone, so that we can develop respectful relationships with no matter who it is. And I think that if you've lived with white privilege, you have no idea of what we have experienced in our life. [. . .] I like to go where my own people are. [. . .] If I know where our people are, like the Ki-Low-Na Friendship Society, I'd rather go there than to go elsewhere*.'	
Smylie et al., 2011 [41]	1. To work in partnership with Aboriginal organizational stakeholders to develop a baseline population health database for urban Aboriginal people living in Ontario that is immediately accessible, useful, and culturally relevant to local, small region, and provincial policy makers	**Main themes:** Poverty, access to stable housing, food security, high need to access emergency departments but poor treatment reported when in emergency departments<br>• 78.2% of the First Nations persons living in Hamilton earn less than $20,000 per year and 70% of the First Nations population in Hamilton lives in the lowest income quartile neighbourhoods compared to 25% of the general Hamilton population<br>• 63% of First Nations community members in Hamilton had to give up important things (i.e. buying groceries) in order to meet shelter-related housing<br>• Identified barriers included long waiting lists (48%), lack of transportation (35%), not able to afford direct costs (32%), doctor not available (29%), and lack of trust in health care provider (24%)<br>• 52% had accessed an emergency department in the past 2 years and 44% of rated the quality of the emergency care as fair or poor<br>• 17% felt availability of health care was excellent, 43% felt it was good, 29 % felt it was fair and 11% felt it was poor	**Main themes:** Strong cultural identity, Culturally appropriate health care, cultural safety,<br>• *'Doctors need to get off their high horse, not just give you the drugs and say, "see you later". Th e ones [doctors] I am with now actually care about people. They follow up and try and help you solve your disorders.'*<br>• *'We need more Aboriginal people in health care, education, places where people are looking up to other people. More native role models.'*

		• *"I need access to a doctor to sign my special diet form so I can lower my cholesterol"*<br>• *'Prejudice'*<br>• *'Lack of trust and cultural understanding'*	
Syme et al., 2011 [40]	1. To explore Aboriginal peoples' experiences of mental health and addictions care in an urban Canadian context to inform the design of safe and effective [mental] health and addiction services.	**Main themes:** Three intersecting issues that impact access to Methadone Maintenance Treatment: stigma and prejudice, social and structural constraints influencing enactment of peoples' agency, and homelessness.<br>• attitude of providers was cited as a barrier to access to care in particular settings by several client and health care professional participants<br>• *'[service providers] don't mean to do it, they don't get up in the morning with a plan to go 'I'm going to go kick ten junkies today,' they don't do it, its just as the day builds, as the day builds they just desensitize, year after year they get desensitized to needs and then they just start dealing with what the immediate needs are.'*	**Main themes:**<br>Not the focus of the study.
Tang et al., 2015 [42]	1. To explore experiences of healthcare access from the perspectives of Indigenous and non-Indigenous patients seeking services in the non-urgent division of an urban Emergency Department (ED) located in a large hospital in a Western Canadian city.	**Main themes:** Racism, discrimination, long waiting times.<br>• *'I was throwing up and for three days, I didn't eat. I told the doctor, I wasn't looking for the drugs, I just couldn't eat and if I eat, I'd throw up. I told the nurse, and she goes, 'well, we need to see that'. I said 'I am not asking for anything, I am just letting you know why I am losing so much weight.'*<br>• We moved to (name of a city in the outskirt) and rented in a nice, well to do neighbourhood, and you are accepted as being normal class, everyday citizens. But as soon as you are known to have come from (name of a downtown core area), the first thing that's thought about you is that you are a drug addict or a drunk, and that's the kind of treatment you get, and it shouldn't be that way . . .You get pigeon-holed and lumped up and stereotyped, and then as soon as I am honest enough to say I have Hepatitis C, the first thing (the healthcare provider asked) is, 'when's the last time you used drugs?' Excuse me, I am not here because I am a drug user; it has been proven that I had Hep C because of all the surgeries I had in my life.	**Main themes:**<br>Not the focus of the study.

		• There are a lot of other people, some of them don't have major injuries but they will go in fast . . . I notice every time I go to see a doctor, I'm waiting for a long time. Like my knee I handled that for about a week and a half before I even decided to go (for treatment), because I knew the waiting time was just going to be a long time, making you wait.	
Tungasuvvingat Inuit, 2017 [43]	1. To share study results and adaptation processes with First Nations, Inuit, and Métis stakeholders in other provinces and territories and thereby contribute to the development of urban Indigenous health data enhancement strategies	**Main themes:** No traditional medicine, cannot understand what the health provider was saying, not comfortable with health provider, costs, cannot afford transportation, health services not available after hours.<br>• Doctor not available after hours (30%)<br>• Nurse not available after hours (16%)<br>• No traditional medicine (31%)<br>• Difficultly getting transportation (22%)<br>• Cannot afford direct costs of car (23%)<br>• Do not have valid Ontario Health Insurance Card (21%)	**Main themes:**<br>Not the focus of the study.
Van Herk et al., 2012 [44]	1. who are Aboriginal people living in the urban setting<br>2. what influences their experiences of accessing care<br>3. what factors influence their access to preventive care.	**Main themes:** Not the focus of this study.	**Main themes:** Safe spaces, safe relational places, belonging and community, engaging the five senses, service provider attitude and commitment,<br>• 'I think what (the Aboriginal-run Center) does really well here is it breaks down the barriers of isolation and provides that community support. Education, you know, we're linkages for people. We did have language classes. Employment, it's about meaningful employment. You'll notice we do try hard to mentor people into roles. Where we'll try and build capacity in our staff, specifically ones who are Aboriginal or of Aboriginal descent. We will move them and see what we can do for them. And we do the same thing with our clients. Anyway good care is about all of those pieces. It is not just about coming into the clinic' (p. 15).<br>• 'I never had much confidence, most of my life, because of the trauma and abuse that I survived. However when I came to [Aboriginal organization], I started to find out who I was and I started to build on my successes. And the reason I built on those

			successes was because I had peer sisters that told me I had gifts and skills where as my mother couldn't build con- fidence within me because she didn't have confidence in herself because she was a product of the residential school Being in a safe place with peer women that work professionally with you but also supported you personally too' (p.3).
	1.	•	
Well Living House, 2016 [45]	1.	**Main themes:** Stigma and discrimination, poverty<br>• 71 of Indigenous adults in Toronto had experienced racism from a health professional that prevented, stopped or delayed from them returning to health services<br>• 72% had experienced unfair treatment more than once in the past 5 years<br>• Over 50% believed their ability to engage in preventative health activities has been affected by financial hardship	**Main themes:**
Wright et al., 2019 [47]	1. To understand how Indigenous mothers typically responsible for the health of their infants—living in urban areas, experience selecting and using health services to meet the health needs of their infants.	**Main themes:** Unwelcoming clinics, poverty.<br>• Mothers suggested that living in these neighborhoods negatively influenced their infant's PCP such that PCPs demonstrated feelings of apathy towards the community by running "ghetto" and unwelcoming clinics<br>• One mother described her experience of attending a rundown clinic in her neighbourhood:<br>o *'…you have to expect living in this area you're not going to get the best healthcare. It seems like they care less when you're in a poverty-stricken area…the doctor's office is kind of ghetto looking. They just threw it together it kind of seems… It doesn't feel personable, it doesn't feel welcoming, it doesn't feel warm, and it feels like you're in and out, and they are not doing their job. They don't ask you how you're doing, as they would in a different nicer area. Ya, I guess in the area you live in you can expect different treatment.'*	**Main themes:** Mothers described four organizational policies that influenced their experiences of using primary care for their infants, including: (a) flexible appointments; (b) alternative options for care; (c) welcoming receptionists; (d) welcoming spaces, (e) multi-service clinics.<br>• mothers who had attended clinics in more affluent neighbourhoods were overwhelmed by spacious clinics that were well-maintained and visually appealing but made them feel out of place.<br>o *'…It was so nice! Like this is going to sound ghetto. Like a high-class pediatrician because they were giving out free baby Tylenol. Right? And my pediatrician doesn't do that for me… a lot of parents when I looked around, you can't help but notice, but they were older, lighter skin parents…They like were more classier looking*

		• some mothers described traumatic experiences of unethical care related to racism and discrimination, which damaged the potential for building trusting relationships. Mothers who experienced racism and discrimination attributed this to their PCP's assumption that they were Indigenous—based on their appearance and/or last name<br>• Primary care providers shared conflicting stories of their ability to provide culturally relevant care. Some providers had taken cultural training in their workplace, while others recognized their need for cultural training to understand of the impacts of colonization and better familiarize themselves of the city's culturally based resources for Indigenous people	*type of people…I felt a little uncomfortable when we were waiting in there.'*<br>• Several mothers expressed that the availability of numerous services within the same facility would improve their experience.<br>• Mothers repeatedly emphasized the importance of having a relationship with their infant's PCP<br>• mothers strongly believed that their infant's PCPs should provide culturally relevant care. They felt this could be accomplished by having an understanding and appreciation of Indigenous history, and by valuing and supporting cultural and spiritual beliefs as they relate to health and well-being
Wylie et al., 2019 [48]	1. This research project examined the perspectives of health care providers and decision makers to identify what challenges they see facing Indigenous patients and families when accessing health services in a large city in southern Ontario.	**Main themes:** unwelcoming environment, stereotyping and stigma, and practice informed by racism.<br>• *'I would say that they have a perception that they are perhaps as not well cared for or as respected when they come in to the facility. Maybe that is a reluctance for them to come and they seek treatment in other places they feel more comfortable with'*<br>• A number of respondents noted that there are times when Indigenous patients come in with expectations of poor treatment, which sets the stage for a challenging interaction with care providers. One respondent explained that they (as a physician) sometimes become defensive when an interaction with an Indigenous patient is not going well.<br>• General frustrations with health care service were also expressed through accusations of discrimination. Other respondents noted similar issues where patients expressed concerns of discrimination they felt were unfounded.<br>• A number of respondents acknowledged their lack of understanding and knowledge of Indigenous issues, culture, and medicinal practices. Instead, they noted that views on Indigenous people are informed by stereotypes perpetuated in the media. One respondent emphasized that systemic discrimination is a significant issue in the health care system that shapes health outcomes	**Main themes:**<br>Not the focus of the study.

		• Respondents noted that physicians commonly blame Indigenous individuals for their health status. Care providers are often unaware of the social determinants of health that have led to poor health outcomes among racialized populations, partly due to the lack of research on this area<br>• Another respondent acknowledged that there is a systemwide belief that Indigenous peoples misuse pain medications, and as a result, Indigenous peoples are not provided adequate pain medication.	
